# MATERNAL AND FETAL RISK FACTORS ASSOCIATED WITH LATE PRETERM INFANTS

**DOI:** 10.1590/1984-0462/2020/38/2018136

**Published:** 2019-11-25

**Authors:** Luísa Krusser Vanin, Helen Zatti, Thaise Soncini, Rodrigo Dias Nunes, Louise Beni Staudt de Siqueira

**Affiliations:** a Universidade do Sul de Santa Catarina, Florianópolis, SC, Brazil.; b Universidade Federal de Santa Catarina, Florianópolis, SC, Brazil.

**Keywords:** Risk factors, Infant, premature, Infant, newborn, Fator de risco, Prematuro, Recém-nascido

## Abstract

**Objective:**

To determine maternal and fetal risk factors associated with the birth of late preterm infants in comparison to those born at term.

**Methods:**

A case-control study was carried out in a tertiary center for high-risk pregnancies. For the cases, the study enrolled post-partum mothers and their respective newborns with gestational ages equal or greater than 34 weeks and less than 37 weeks. As controls, the post-partum mothers and their newborns with gestational ages of 37 weeks or greater were selected. The sample was calculated with a ratio of two controls for each case, resulting in 423 patients. Association studies were performed using the chi-square test or Fisher’s exact test and logistic regression analysis.

**Results:**

The variables associated with late prematurity were inadequate prenatal (*Odds Ratio* [OR] 1.23; confidence interval of 95% [95%CI] 1.12-1.34; p≤0.001), premature rupture of membranes (OR 4.98; 95%CI 2.66-9.31; p≤0.001), length of hospital stay ≥24 hours until birth (OR 0.18; 95%CI 0.06-0.52; p≤0.001), cesarean section (OR 2.74; 95%CI 1.69-4.44; p≤0.001) and small for gestational age newborn (OR 3.02; 95%CI 1.80-5.05; p≤0.001).

**Conclusions:**

Inadequate prenatal care and membranes’ premature rupture were found as factors associated with the late preterm birth. It is important to identify the factors that allow intervention with adequate prenatal care in order to reduce poor outcomes due to late prematurity.

## RESUMO

**Objetivo:**

Determinar fatores maternos e fetais associados ao nascimento de recém-nascidos prematuros tardios, quando comparados aos nascidos a termo.

**Métodos:**

Estudo caso-controle em um hospital terciário de referência para atendimento de gestações de alto risco. Foram considerados casos as puérperas e seus respectivos recém-nascidos com idade gestacional maior ou igual a 34 semanas e menor de 37 semanas. Para os controles foram selecionadas as puérperas e seus recém-nascidos com idade gestacional de 37 semanas completas ou mais. A amostra foi calculada com razão de dois controles para cada caso, resultando em um total de 423 pacientes. Estudos de associação foram efetuados utilizando-se o teste do qui-quadrado ou teste exato de Fisher e posterior regressão logística.

**Resultados:**

As variáveis associadas à prematuridade tardia foram a realização de pré-natal inadequado (*Odds Ratio* - OR 1,23; intervalo de confiança de 95% - IC95% 1,12-1,34; p≤0,001), a rotura prematura de membranas amnióticas (OR 4,98; IC95% 2,66-9,31; p≤0,001), o tempo de internação ≥24 horas até o nascimento (OR 0,18; IC95% 0,06-0,52; p≤0,001), o parto operatório (OR 2,74; IC95% 1,69-4,44; p≤0,001) e o recém-nascido pequeno para a idade gestacional (OR 3,02; IC95% 1,80-5,05; p≤0,001).

**Conclusões:**

Assistência pré-natal inadequada e rotura prematura de membranas destacaram-se como fatores associados ao nascimento de prematuros tardios. Ressalta-se a relevância da identificação de fatores passíveis de intervenção por meio de adequada assistência pré-natal, a fim de reduzir os desfechos desfavoráveis decorrentes da prematuridade tardia.

## INTRODUCTION

According to the World Health Organization (WHO), the definition of prematurity encompasses all newborns (RN) born before 37 weeks of gestational age (GA). Late premature infants are those born between 34 and 36 weeks and 6 days, and extreme premature infants are born before 28 weeks of gestational age.^1^ Prematurity occurs in more than one in ten births, and it is the leading cause of morbidity and mortality among newborns, with about 1.1 million deaths per year worldwide. Of these deaths, most can be prevented with basic care and low-cost interventions.^1^ ^,^ ^2^

The main maternal complications that lead to late preterm birth are premature labor (PL) and premature rupture of membranes (PROM). Other obstetric conditions contribute to prematurity, such as urinary tract infection, hypertensive disease, gestational diabetes, and twin pregnancies. Among the main fetal factors are restricted intrauterine growth and the non-reassuring fetal status.^3^ ^,^ ^4^ ^,^ ^5^ ^,^ ^6^

The risk of neonatal complications is inversely proportional to GA. Each week the fetus remains in the womb, its development improves and the frequency and severity of neonatal complications are improved. Early complications of late prematurity include respiratory distress syndrome, apnea, hypothermia, hypoglycemia, hyperbilirubinemia, eating difficulties, central nervous system immaturity, and infections, with a risk of at least seven times more complications in this group compared to term infants (TI). ^6^ ^,^ ^7^ ^,^ ^8^ Morbidity and risk of hospitalization during the first year of life are also higher compared to term infants.^8^ ^,^ ^9^ Long-term studies indicate that, when compared to preterm infants, late preterm infants (LPI) are at an increased risk for neurodevelopmental disorders and learning disabilities, with neurocognitive changes that may persist into adulthood.^8^ ^,^ ^10^ ^,^ ^11^ ^,^ ^12^ ^,^ ^13^

Because extreme preterm infants receive greater attention from health professionals for their high risk of complications, LPI infants are often neglected and treated the same as TI, which increases their risk for complications. ^4^ ^,^ ^14^ ^,^ ^15^ ^,^ ^16^ Therefore, the term “late premature” rather than “near term” is recommended in order to avoid the misinterpretation that preterm infants that are born close to term, share the same risks as a TI.^1^ ^,^ ^17^

Knowledge of the factors that lead to the birth of late preterm infants can provide subsidies for health professionals and managers to prevent them, and reduce neonatal morbidity and mortality related to prematurity. Thus, this study aimed to identify factors associated with the birth of LPIs.

## METHOD

This is a case-control study developed at the maternity hospital of a tertiary reference hospital for high-risk pregnancies, which exclusively serves the Public Health System (*Sistema Único de Saúde* - SUS) in the state of Santa Catarina. The population was composed of patients whose deliveries were performed at the hospital, from January to June 2016, until the calculated sample size was reached.

We included in the case group puerperal women and their respective newborns with a GA greater than or equal to 34 weeks and less than 37 weeks. The control group included postpartum women and their newborns with a GA of 37 weeks or older. GA was defined using an obstetric ultrasound (when performed up to 20 weeks of gestation), the date of the woman’s last menstruation or somatic Capurro after birth. This method was preferred over calculating the date of the last menstruation when the difference between the two methods was greater than two weeks.

The medical records of interest were selected from the hospital’s birth records and the sample selection was consecutive, including all preterm births until the sample size was completed. For each LPI, the two subsequently born TI were included. Cases of fetal deaths and those with major malformations were excluded.

The sample was calculated with a confidence interval of 95% (95%CI), a statistical power of 80%, and a ratio of two controls for each case, estimating an average prevalence of the various risk factors assessed from 15% of the controls and an Odds Ratio (OR) of at least double. The procedure resulted in a final sample of 423 patients (141 cases and 282 controls). The independent variables of the study were sociodemographic, clinical-obstetric and perinatal characteristics. The variable maternal hypertensive disease included all of the high blood pressure conditions diagnosed in the pregnant woman, such as chronic hypertension, pregnancy-specific hypertensive disease, preeclampsia and eclampsia. For variable diabetes, we considered the mothers with a previous diagnosis of diabetes mellitus types 1, 2 or gestational. Prenatal care was considered to be inadequate when there were five or fewer medical visits during pregnancy, regardless of GA. PROM was diagnosed when it occurred after the 20th week of pregnancy and before the onset of labor. Bacterial infection was considered only when it was present during pregnancy, and included urinary tract infection and chorioamnionitis.

First, the independent variables were described in absolute numbers and proportions, according to the outcome (late prematurity). The Chi-square test or Fisher’s exact test was used to test the homogeneity of the proportions. Stratified analysis was used to obtain an association between two variables. For the multivariate logistic regression analysis, the forward stepwise method was used to investigate the independent effect of the variables that were potentially associated with the outcome. To enter the variables in the logistic regression model, those with p> 0.05 were removed. However, a non-significant variable remained and, removing it from the model, we arrived at the final model with only five predictor variables. Thus, only variables with p≤0.001 were entered. The Statistical Package for the Social Sciences (SPSS) 18.0 (Chicago, IL) program was used in the statistical analysis of the data. The magnitude of the association between late prematurity and independent variables was measured by OR and it had the respective 95%CI.

The study complied with all applicable ethical criteria and was approved by the Research Ethics Committee of the Universidade do Sul de Santa Catarina (CEP-Unisul) under CAAE 62363316.0.0000.5369. The risks inherent in this research were minimal, since the analysis of medical records was performed and the privacy and anonymity of the information collected was ensured.

## RESULTS

Information on mothers and their respective newborns were obtained from 423 forms. There were 141 cases of late preterm infants and 282 term infants. Among the maternal sociodemographic characteristics when considering the total sample, age ranged from 14 to 44 years old, with an average age of 26 years. Additionally, 49.2% of the parturients had up to nine years of schooling. Mothers in the higher gestational risk age groups (≤15 or ≥35 years old) were observed in 22.7% of the mothers in the case group and 15.3% in the control group, but there was no significant difference. Of these pregnancies, 1.7% were twins, and all of them were premature births. The clinical and sociodemographic characteristics of the postpartum women are available in Table 1.

**Table 1:** Clinical and sociodemographic characteristics of puerperal women with late preterm and term infants.

	LPI		TI		p-value
	n	(%)	n	(%)	
Age (years)					
≤15	4	(2.8)	7	(2.5)	
16-34	109	(77.3)	238	(84.7)	0.953
≥35	28	(19.9)	36	(12.8)	0.671
Schooling (years)					
Up to 9	71	(50.4)	137	(48.6)	0.603
From 9 to 12	59	(41.8)	119	(42.2)	0.686
More than 12	11	(7.8)	26	(9.2)	
Hypertensive disease					
Yes	21	(14.9)	27	(9.6)	0.104
No	120	(85.1)	255	(90.4)	
Diabetes					
Yes	8	(5.7)	8	(2.8)	0.149
No	133	(94.3)	255	(97.2)	
Prenatal					
Inadequate	56	(39.7)	65	(23.1)	<0.001
Adequate	85	(60.3)	217	(76.9)	
Start of pre-natal care					
1^st^ trimester	130	(92.2)	253	(89.7)	0.215
2^nd^-3^rd^ trimester	11	(7.8)	29	(10.3)	
PROM					
Yes	42	(29.8)	24	(8.5)	<0.001
No	99	(70.2)	258	(91.5)	
Bacterial infection					
Yes	10	(7.1)	22	(7.8)	0.794
No	131	(92.9)	260	(92.2)	
Length of hospital stay					
≥ 24 hours	15	(10.6)	4	(1.4)	<0.001
< 24 hours	126	(89.4)	278	(98.6)	
Delivery route					
Cesarean	69	(48.9)	86	(30.6)	<0.001
Vaginal	72	(51.1)	196	(69.4)	

LPI: late premature infant; TI: term infant; PROM: premature rupture of the membranes.

By analyzing the cases and controls together in relation to maternal habits and diseases, 7.5% of pregnant women had bacterial infection at the time of delivery, and 91.3% had a urinary tract infection. There were STORCH infections (syphilis, toxoplasmosis, rubella, mumps, herpes, among others) in 6.9% of the pregnancies. Syphilis was the most frequent infection, accounting for 48.1% of STORCH-type infections and present in 3.1% of the total sample. It was observed that 14.2% of pregnant women used (legal or illegal) drugs during pregnancy. The most frequent was cigarettes. Other drugs used were crack, marijuana and alcohol.

Regarding clinical and obstetric history, 29.8% of the pregnant women, both in the case and the control groups, were nulliparous. 89.3% of all pregnant women started prenatal care in the first trimester of pregnancy, but only 71.4% had a minimum of six prenatal visits. In the group of pregnant women with LPI, 60.3% went to the minimum number of consultations, while the number of pregnant women with TI was significantly higher at 76.9% (crude OR 2.20; 95%CI 1.41-3.40; p<0.001). Nine pregnant women did not attend prenatal consultations, seven of them were mothers of preterm infants, resulting in 5% of pregnant women with preterm births without any prenatal care. Among the case group pregnant women, PL (23.4%) and PROM (29.8%) accounted for more than half of spontaneous preterm births. Significance was also observed when comparing the mode of delivery, in which 69.4% of full-term and only 51.1% of late preterm infants were vaginally born (crude OR 2.18; 95%CI 1.43-3.31; p<0.001). Intrauterine growth restriction (IUGR) was diagnosed in eight pregnant women with LPI (5.7%) and in three with TI (1.1%).

The birth weight of preterm infants ranged from 1800 to 4230 g, with a median of 2697 g. 24.1% of them were small for gestational age (SGA). Among TI, birth weight ranged from 2170 to 5030 g, with a median of 3366 g. After adjusting the OR value and the p value using logistic regression, the gender variable of the newborn was no longer statistically significant (Tables 2 and 4).

**Table 2:** Comparative analysis between fetal and neonatal characteristics with late preterm and term birth.

	LPI		TI		p-value
	n	(%)	n	(%)	
Small for GA					
Yes	34	(24.1)	22	(7.8)	<0.001
No	107	(75.9)	260	(92.2)	
Large for GA					
Yes	5	(3.5)	36	(12.8)	<0.001
No	136	(96.5)	246	(87.2)	
Gender					
Male	64	(45.4)	157	(55.9)	0.042
Female	77	(54.6)	124	(44.1)	

LPI: late premature infant; TI: term infant; GA: gestational age.

In adjusting for the confounding effect of covariates in the multivariate model described in Tables 3 and 4, time between hospitalization and delivery and SGA birth, the association with late prematurity remained significant, even though the effect had fallen in importance for adequate prenatal care. For the other variables of the multivariate model, the chance of PROM and operative delivery increased in the group of late preterm infants.

**Table 3:** Bivariate and multiple logistic regression between maternal risk factors for prematurity.

	Crude OR (95%CI)		p-value^‡^	Adjusted OR (95%CI)	p-value^§^
Age (years)					
≤15	1			#	
16-34	0.80	(0.22-3.18)	0.953		
≥35	1.36	(0.30-6.96)	0.671		
Schooling (years)					
Up to 9	1.22	(0.57-2.71)	0.603	#	
From 9 to 12	1.17	(0.54-2.62)	0.686		
More than 12	1				
Hypertensive disease					
Yes	1.65	(0.88-3.04)	0.104	#	
No	1				
Diabetes					
Yes	2.06	(0.72-5.79)	0.149	#	
No	1				
Pre-natal					
Inadequate	2.20	(1.41-3.40)	<0.001	1.23 (1.12-1.34)	<0.001
Adequate	1			1	
Start of pre-natal care					
1^st^ trimester	0.61	(0.26-1.30)	0.2147	#	
2^nd^-3^rd^ trimester	1				
PROM					
Yes	4.54	(2.62-7.98)	<0.001	4.98 (2.66-9.31)	<0.0001
No	1			1	
Bacterial infection					
Yes	0.90	(0.39-1.94)	0.794	#	
No	1				
Length of hospital stay					
≥ 24 hours	8.23	(2.80-29.36)	<0.001	0.18 (0.06-0.52)	<0.001
< 24 hours	1			1	
Delivery route					
Cesarean	2.18	(1.43-3.31)	<0.001	2.74 (1.69-4.44)	<0.001
Vaginal	1			1	

Adjusted OR: *Odds Ratio* adjusted for logistic regression; PROM: premature rupture of the membranes; ^‡^crude p; ^§^p adjusted by other model variables.

**Table 4:** Bivariate and multiple logistic regression between fetal and neonatal risk factors for prematurity.

	Crude OR (95%CI)		**p*-*valor** ^†^	Adjusted OR (95%CI)	p-value^‡^
Small for GA^*^					
Yes	3.742	(2.09-6.77)	<0.001	3.018 (1.80-5.05)	<0.001
No	1				
Gender					
Male	0.436	(1.01-2.27)	0.042	#	
Female	1				

Crude OR: Crude Odds Ratio; Adjusted OR: Odds Ratio adjusted by logistic regression; GA: gestational age; † crude p; ‡ p adjusted by other model variables; *live births with birth weight below the 10^th^ percentile according to gestational age.

## DISCUSSION

The prematurity rate in Brazil is 11.5%, almost twice that observed in European countries, with 74% of these premature infants being late.^18^ The present study analyzed the factors associated with late prematurity, finding a significant number of biological and social determinants that need to be known and identified early in order to avoid it.

Studies in Canada ^14^ and Jordan^15^ describe lower levels of maternal education in addition to low socioeconomic status, as an independent causal factor for premature birth and increased neonatal morbidity. In the present study, however, there was no association between low levels of maternal education and late prematurity. This fact may be due, in part, to the impossibility of stratifying the group with up to nine years of schooling. Furthermore, indicators of socioeconomic level were not evaluated.

Several authors^4^ ^,^ ^6^ ^,^ ^16^ found a significant association between the presence of maternal comorbidities and the risk of prematurity. The main comorbidities were diabetes and maternal hypertensive disease. This was described as the condition that most often caused premature birth in these studies. There was no statistically significant association between premature birth and the presence of maternal hypertensive disease or diabetes in the study group. It is possible that this finding may be due to the lower incidence of maternal hypertensive disease in the study population or that the sample was not large enough to detect such associations.

Several studies relate prematurity with inadequate prenatal care.^16^ ^,^ ^19^ ^,^ ^20^ In the study by Nadin et al.,^15^ an almost four times higher risk of premature birth was found in women that did not receive prenatal care. The risk was significantly lower in the group that went through prenatal visits. The more consultations attended, the lower the frequency of premature births. In the present study, incomplete prenatal care was a predictor of late prematurity, which is consistent with what was described by Machado et al. ^21^ in a study conducted in southern Brazil. In the present study, the number of pregnant women with incomplete prenatal care was extremely high, and in the group of late preterm infants, 39.7% of pregnant women had less than six prenatal consultations, a number far above the 18.6% described by Machado et al.^21^ It should be emphasized, however, that a smaller number of consultations are expected in prematurely terminated pregnancies compared to term pregnancies, since the pregnant woman spend less time being pregnant. However, one third of women that have delivered prematurely through SUS have had fewer consultations than recommended for GA. This information ^20^ ^,^ ^22^ may have an impact on the high prevalence of premature births in Brazil, which is in tenth position among countries with the highest absolute number of premature births.^20^

The most frequent maternal infection present during childbirth was a urinary tract infection, which, according to the Brazilian Multicenter Study on Preterm Birth, is related to PL and prematurity. In the present study, however, it was similar in the case and control groups, which may be attributed to the treatment of infections.

In the study performed by Laughon et al.,^4^ spontaneous preterm birth, including PL and PROM, accounted for about two thirds of all late preterm births. The other third was represented by other identifiable risk factors, such as hypertensive disease, diabetes and non-tranquilizing fetal status (signs suggestive of fetal homeostasis loss). A small percentage did not have an identifiable risk factor and could have resulted from underreporting of maternal or fetal conditions, or from the choice to have surgery. In the current study, PL and PROM accounted for 53.2% of premature births, which is similar to the percentage reported in the current literature. ^4^ ^,^ ^6^ ^,^ ^19^

IUGR was diagnosed in 5.7% of LPI, while 24.1% of newborns in the same group were classified as SGA. The variable of low weight for GA was chosen to assess the correlation with premature birth, since IUGR may have been under diagnosed in this population due to prenatal deficiencies. It is known that when using the SGA variable, a portion of constitutionally small newborns is included. On the other hand, cases where growth decelerated, but did not end in SGA, may have been excluded. Nevertheless, many studies use SGA as a substitute for IUGR due to the difficulty in diagnosing it. ^23^ ^,^ ^24^ ^,^ ^25^ ^,^ ^26^ In addition, SGA LPI represent a high-risk group for short- and long-term adverse outcomes and may be associated with even higher mortality. Regarding the gender of the newborn, although initial statistical significance was obtained with p≤0.05 in the bivariate analysis, this variable was not included in the logistic regression due to the methodological decision to select only those with p ≤0.001.

When maternal or obstetric complications occur, the risks and benefits of the delivery method should be considered with expectant management for maternal, fetal and neonatal health in order to determine the best time for delivery. The American study by Nadin et al.^15^ showed that early maternal hospitalization during pregnancy indicates the existence of potential maternal or fetal health problems, which may either cause premature birth or indicate a caesarean section. Thus, in the present study, there was a higher frequency in the group of pregnant women with preterm infants of patients who were hospitalized for 24 hours or more until delivery, probably because there was an attempt made to delay premature birth by inhibiting spontaneous labor or compensate for any existing maternal pathology.

Since 1985, the WHO has stated that caesarean sections are an effective life-saving intervention for mothers and babies, but only when indicated for medical reasons. Caesarean section rates greater than 10-15% are not associated with reduced maternal and neonatal mortality. According to 2014 data, Brazil has a high prevalence of operative births (52.8%), with 38.1% being performed in public hospitals.^28^ ^,^ ^29^ These figures are consistent with the findings of the present study, in which the cesarean section rate was 48.9% in LPI and 30.6% in TI. However, the variable for recommended cesarean delivery was not evaluated, thus limiting the analysis of the high rate found, which can be justified due to the obstetric characteristics attended. The studied hospital has a reference maternity ward for the care of high-risk pregnancies in SUS, where the most complex cases are referred. These are cases where the likelihood of needing a caesarean section is great. An ecological study^30^ conducted with data from developed countries showed that where there were higher rates of prematurity, fetal and neonatal death rates were lower. The authors’ argument is that deliveries that happened for medical reasons with preterm infants are generally beneficial because they were performed on those most at risk of death. Preventive strategies to reduce iatrogenic preterm birth, especially in late preterm pregnancy, should carefully consider the underlying obstetric risks and the high-risk pregnancies’ need for medical intervention.

Among the limitations of this study, we highlight the fact that data obtained from medical records were used, which means that there was a lack of information about some variables, preventing the stratification of certain characteristics. In addition, due to the study design, it is not possible to assess the prevalence of late prematurity, as there is an increased chance of bias by retrospectively measuring the predictor variables. However, the presence of a control group allowed for the identification of factors associated with early delivery, not just a description of the characteristics of late preterm infants. It was also chosen to assume an extremely low significance value, thus minimizing the chance of causality of the results.

Since late prematurity is an independent risk factor for neonatal morbidity when compared to TIs,^12^ knowing the particularities of each GA and identifying the risk factors that can be addressed, are important. They allow the obstetrician and the pediatrician to provide appropriate care and advice to the patient, considering that there are different approaches to be taken in each of the groups.^4^

Among the main determinants associated with late preterm birth in this study are incomplete prenatal care and PROM. Other associated factors were length of hospitalization until birth greater than or equal to one day, operative delivery and SGA newborn. These factors, especially the high number of late preterm births that did not receive adequate prenatal care, show the importance of health policies that can include this group of pregnant women, thus reducing unfavorable outcomes. This study may contribute to the improvement of health policies in our country by identifying maternal and perinatal factors associated with late prematurity. These factors highlight the need to improve access and quality of prenatal care, thus reducing the number of late preterm births and the significance it represents for health
